# D-Alpha-Tocopheryl Poly(ethylene Glycol 1000) Succinate-Coated Manganese-Zinc Ferrite Nanomaterials for a Dual-Mode Magnetic Resonance Imaging Contrast Agent and Hyperthermia Treatments

**DOI:** 10.3390/pharmaceutics14051000

**Published:** 2022-05-06

**Authors:** Lin Wang, Syu-Ming Lai, Cun-Zhao Li, Hsiu-Ping Yu, Parthiban Venkatesan, Ping-Shan Lai

**Affiliations:** 1 College of Chemistry & Pharmacy, Northwest A&F University, Xianyang 712100, China; wanglin0317@nwsuaf.edu.cn (L.W.); hsiupingyu0126@gmail.com (H.-P.Y.); 2 Department of Chemistry, National Chung Hsing University, Taichung 402204, Taiwan; btx600@gmail.com (S.-M.L.); hjp65l4@hotmail.com (C.-Z.L.); venkatesanwala@gmail.com (P.V.); 3 Ph.D. Program in Tissue Engineering and Regenerative Medicine, National Chung Hsing University, Taichung 402204, Taiwan

**Keywords:** theranostic, manganese-zinc ferrite, magnetic resonance imaging, hyperthermia, in vivo

## Abstract

Manganese-zinc ferrite (MZF) is known as high-performance magnetic material and has been used in many fields and development. In the biomedical applications, the biocompatible MZF formulation attracted much attention. In this study, water-soluble amphiphilic vitamin E (TPGS, d-alpha-tocopheryl poly(ethylene glycol 1000) succinate) formulated MZF nanoparticles were synthesized to serve as both a magnetic resonance imaging (MRI) contrast agent and a vehicle for creating magnetically induced hyperthermia against cancer. The MZF nanoparticles were synthesized from a metallic acetylacetonate in an organic phase and further modified with TPGS using an emulsion and solvent-evaporation method. The resulting TPGS-modified MZF nanoparticles exhibited a dual-contrast ability, with a longitudinal relaxivity (35.22 s^−1^ mM Fe^−1^) and transverse relaxivity (237.94 s^−1^ mM Fe^−1^) that were both higher than Resovist^®^. Furthermore, the TPGS-assisted MZF formulation can be used for hyperthermia treatment to successfully suppress cell viability and tumor growth after applying an alternating current (AC) electromagnetic field at lower amplitude. Thus, the TPGS-assisted MZF theranostics can not only be applied as a potential contrast agent for MRI but also has potential for use in hyperthermia treatments.

## 1. Introduction

Cancer treatment is an important issue in biomedical research, and a tremendous amount of effort has been devoted to enhancing the efficiency of diagnosis and therapy. Magnetic nanoparticles provide not only specific material properties but also a combination platform for imaging, therapy, and targeting, making them an ideal theranostic (therapy and diagnosis) agent. Iron oxide-based nanoparticles were the first magnetic materials used, but there are different types of magnetic materials, including MFe_2_O_4_ ferrites (M = Mn, Ni, Co, Zn, Fe, and other divalent cations), that have attracted interest because of their intriguing magnetic properties. Studies of iron oxide and metal ferrites have been published, and these materials are widely used in biomedical applications for multifunction theranosis [1,2,3].

MFe_2_O_4_ nanoparticles are of significant interest because of their intrinsic biocompatibility, tunable magnetic characteristics, higher transition temperature, and superior chemical stability for room temperature applications due to the presence of cations. Magnetic hyperthermia and MRI contrast have recently been described using MFe_2_O_4_ nanoparticles [4]. In the case of magnetic hyperthermia, high values of the specific absorption rate (SAR) are required to generate an adequate temperature increase, which is achieved by administering as few nanoparticles as possible. To improve the SAR, various aspects such as composition, shape, and structure were evaluated. Several studies have shown that the addition of Mn and Zn to the magnetic nanoparticle structure, which optimizes the nanoparticle composition, results in higher SAR values than other doping elements, such as nickel or cobalt. Furthermore, by changing the composition of the material, the magnetic properties of Mn-Zn ferrites (i.e., saturation magnetization and magnetic susceptibility) can be tuned and controlled [5]. Iron oxide-based nanoparticles with strong magnetic moment are mainly perform as the *T_2_*-weighted MRI contrast agents in the applications clinical MR imaging [6,7]. The high ratio of transverse to longitudinal relaxivity (*r_2_/r_1_*) of iron oxide nanoparticles limits their utility as *T_1_* contrast agents [8]. Gd^3+^, Mn^2+^, and Fe^3+^ are commonly employed as *T_1_* contrast agents [9,10,11]. The *T_1_* contrast effect is induced by proton interactions with electron spins of contrast agents. However, Gadolinium complex based *T_1_* contrast agent are commercially available the MRI contrast agents are associated to the formation of nephrogenic systemic fibrosis (NSF) [12]. Ever since MRI began, the manganese (Mn) has been used as a positive contrast agent in developing gadolinium-free *T_1_* contrast agents. Mn-based contrast agents might track cells more precisely than Gd-based contrast agents with less toxicity [13,14,15]. They are highly magnetized and highly relaxed due to their huge magnetic spin magnitude. The development of Zn-based MRI contrast agents could be a valuable tool because Zn binding increases transverse relaxivity [16,17,18]. In comparison to iron oxide-based contrast agents, MZF nanoparticles have better biocompatibility, chemical stability, and aqueous suspension than iron oxide-based contrast agents [19,20,21,22]. 

Notably, there were various ionic sufactans such as sodium dodecyl sulfate, poly-ethyleneimin, and cetyltrimethylammonium bromide have been employed as coating agents for magnetic materials; however, these ionic surfactants are quite toxic to cells. The less cytotoxicity PEG-based materials also have been employed as emulsifiers or stabilisers to form water-dispersed spherical cluster [23]. The d-tocopheryl polyethylene glycol 1000 succinate (TPGS) is a water-soluble PEG derivative of natural vitamin E with amphiphilic structure. Due to its bulky structure and large surface area, TPGS performed as a good emulsifier, solubilizer, and stabilizer of hydrophobic medicines. TPGS can also reduce P-glycoprotein related multidrug resistance in cancer cells, improve drug absorption, and increase cytotoxicity and oral bioavailability of anticancer medicines [24]. The vitamin E in TPGS also provide a potential antioxidient ability for metal-medialted oxidiative stress. In this study, we demonstrated a simple procedure for fabricating MZF formulations through the method of emulsion and solvent-evaporation using nonionic polymers that provide not only dual *T_1_*/*T_2_* imaging contrast enhancements but also magnetic field stimulated hyperthermia compared with previous iron oxide-based *T_1_* MR imaging contrast/hyperthermia nano theranostics.

## 2. Materials and Methods

### 2.1. Materials

Iron (III) acetylacetonate (Fe(acac)_3_), manganese (II) acetylacetonate (Mn(acac)_2_), zinc (II) acetylacetonate (Zn(acac)_2_), K_4_Fe(CN)_6_·3H_2_O, dimethylsulfoxide (DMSO), 1,2-hexadecanediol, antibotics penicillin-streptomycin-neomycin solution, 3-(4,5-Dimethylthiazol-2-yl)-2,5-diphenyltetrazolium bromide (MTT) and 10% formalin were purchased from Sigma-Aldrich (St. Louis, MO, USA). Dibenzyl ether and oleylamine were purchased from ACROS (Newark, NJ, USA) and vitamin E TPGS 1000 was purchased from BASF. The oleic acid (99%) was obtained from Showa (Tokyo, Japan), and hydrochloric acid was purchased from UCW (Hsinchu, Taiwan). Modified Eagle’s Medium (MEM), fetal bovine serum (FBS) and 0.25% trypsin-EDTA were obtained from Gibco (Gibco-BRL, Waltham, MA, USA) for cell culture use. Double-distilled water was used in all of the aqueous solutions.

### 2.2. Synthesis of Manganese Zinc Ferrite Nanoparticles

Manganese zinc ferrite nanoparticles were synthesized through a modified procedure based on thermal decomposition reaction under a nitrogen condition, a concept inspired by previous reports [22,25]. The treated Mn^2+^ cation concentration of MZF nanoparticles (Mn_x_Zn_1−x_Fe_2_O_4_) in synthesis process was systematically controlled in x = 0.2, 0.5, 0.8, respectively. In brief, the Mn(acac)_2_ (0.2 mmole, 0.5 mmole, 0.8 mmole, respectively), Zn(acac)_2_ (0.8 mmole, 0.5 mmole, 0.2 mmole, respectively), Fe(acac)_3_ (2 mmole) and 1,2-hexadecanediol (2 mmole) were mixed with dibenzyl ether (20 mL) at 60 °C in a three-neck round-bottomed flask equipped with a condenser, magnetic stirrer, thermograph, and heating mantle and stirred under nitrogen. The surfactants oleic acid about 1 mmole and oleylamine about 1 mmole were then added to the solution. After increasing temperature at a rate of 5 °C/min and keeping at 200 °C for 10 min, the solution was further heated at a rate of 2.5 °C/min up to 285 °C for refluxing and kept for another 30 min. After cooling to room temperature about 25–28 °C, the product MZF was collected by using excess ethanol for precipitation and followed by centrifuged at 6000 rpm for 5 min. After vacuum dried, the collected MZF were then characterized with a vibration sample magnetometer (VSM, LakeShore 735, Westerville, OH, USA) and Powder X-ray Diffractometer (XRD, PANalytical X’Pert Pro MRD, Malvern Panalytical, WR14 1XZ, Malvern, UK).

### 2.3. Prepartation and Characterization of MZF@TPGS Formulatoin

The MZF@TPGS formulations were prepared according to our previous report with modification [26]. Briefly, dried MZF (5 mg) was re-dispersed in 0.2 mL of hexane with 10 μL of oleic acid at room temperature, and the 10 mL of 2 mg/mL TPGS solution was added to the mixture with 10 min sonication to form an emulsion. Solvent was removed by non-magnetic stirring at 1000 rpm on a heating mantle at 85 °C to prevent MZF nanoparticle aggregation on the magnetic stir bar. After cooling down to room temperature, the MZF@TPGS solution was placed under an external magnetic field to remove free TPGS.

The size distribution of the MZF@TPGS formulation was analyzed by dynamic light scattering (DLS, ZS 90, Malvern Panalytical, WR14 1XZ, Malvern, UK) at 25 °C. The samples for TEM analysis were prepared by spreading a drop of the diluted MZF@TPGS formulation solution on carbon-coated 200 mesh copper grids. The samples were dried in air and then observed with a transmission electron microscope (TEM, JEM 1400, JEOL Ltd., Tokyo, Japan) with an accelerating voltage of 120 kV. 

Iron concentrations in the MZF@TPGS was analyzed by atomic absorbance spectrophotometer (AAS, GBC 932, Dandenong, VIC, Australia) and magnetic properties of MZF@TPGS formulations were evaluated using the VSM at room temperature (~300 K). In the hyperthermia test, the AC electromagnetic field induced temperature change of the MZF@TPGS formulations was measured using an AC solenoid coil-capacitor system. The iron concentration of the MZF@TPGS formulations for measuring the heating temperature was fixed at 0.4 mg Fe/mL, and the applied frequency was set by a function generator to 100 kHz with 10 Oe for 30 min. The started temperature 32 °C was controlled at beginning before the demonstration of hyperthermia. The temperature was recorded by an IR thermometer (MS LT, Optris, Berlin, Germany).

To evaluate the magnetic relaxivity, the MZF@TPGS solution was serially diluted and placed into tubes. A 0.47 T (20 MHz) Bruker Minispec mq20 MR Analyzer was used to measure the longitudinal and transverse relaxation times (*T_1_* and *T_2_*, respectively) of aqueous solutions of the MZF@TPGS formulations at 37 °C. The *T_1_*-weighted and *T_2_*-weighted MR contrast agent abilities were evaluated on a 3 T clinical magnetic resonance imaging system (MRI, Signa Excite 3 T, GE Healthcare, Chicago, IL, USA). The samples were placed into 8-channel head coil with a homemade water tank. *T_1_*-weighted two-dimensional imaging were used fast spin echo pulse sequences (TR/TE = 550/13 ms). The field of view (FOV) was 14 cm × 10 cm with slice thickness 1.0 mm and 0.5 mm per gap. *T_2_*-weighted two-dimensional imaging was applied fast-spin echo pulse sequence (TR/TE = 3017/98.9). The field of view was 14 × 7 cm with slice thickness 1.0 mm and 0.5 mm per gap. The MR images were further analyzed on the imaging software (GE Healthcare, Advantage Workstation 4.207).

### 2.4. Cell Cytotoxicity and Cellular Uptake of MZF@TPGS In Vitro

In the following biologically evaluations, the as-prepared MZF@TPGS were lyophilized. The dried sample of MZF@TPGS were diluted into PBS buffer and filtered with 0.22 μm nylon filter, the iron concentration of filtered MZF@TPGS formulations in PBS solution was analyzed by AAS before in vitro studies. KB cells were grown with minimum essential medium (MEM) with 10% fetal bovine serum (FBS) and penicillin-streptomycin-neomycin (1%) in a humidified atmosphere containing 5% CO_2_ at 37 °C. The cells were sub-cultured about three times a week. To evaluate the cytotoxicity of the MZF@TPGS formulations, 8000 cells per well KB cells were seeded into 96-well plates. After 24 h, the old culture medium was discarded and the dilute MZF@TPGS formulations were diluted with medium then added 0.1 mL into wells with two-fold serial dilutions. After another 24 h incubation, the old culture medium with formulations was discarded washed twice with PBS. In the hyperthermia test group, the cells were treated the MZF@TPGS formulations for 24 h and controlled at 32 °C as the started temperature, after which time treatment with an AC solenoid coil-capacitor system operating at 100 kHz and approximately 10 Oe was performed for 30 min, followed by incubation for 24 h. At the end of the incubation, 100 µL of MTT solution (0.5 mg/mL) was added to wells and incubated for 2 h. The solution was gently removed and dimethyl sulfoxide (DMSO) was used to dissolve the formazan. The absorbance was measured at 485 nm using a scanning multiwell ELISA reader (SpectraMax^®^ M2e, Molecular Devices, San Jose, CA, USA) [27]. 

The cellular uptake of the MZF@TPGS formulations was observed by the Prussian blue staining. The 20,000 KB cells were cultured into 3.5 cm dishes with 24 h incubation. The MZF@TPGS formulations with a 20 μg Fe/mL concentration diluted with medium were incubated with cells for different incubation time. Cells were then washed with PBS and fixed with 3.7% formaldehyde PBS solution for 30 min. The internalized MZF nanoparticles were visualized by Prussian blue staining and cells were counterstained with Nuclear Fast Red (NFR, Sigma Aldrich, St. Louis, MO, USA) [28] and observed by light microscopy (DM IL, Leica, Wetzlar, Germany) with imaged by Canon camera (550D). 

### 2.5. In Vivo MR Imaging

In this study, all in vivo experiments were reviewed and approved by the Institutional Animal Care and Use Committee of National Chung Hsing University (IACUC of NCHU), the ethic approval number: IACUC No. 102-127^R^(2013). The age of 3–4 weeks with weight about 19 ± 2 g BALB/cAnN.Cg-*Foxn1^nu^*/CrlNarl nude mice were purchased from National Laboratory Animal Center (Taiwan). All of the experimental mice were kept in an artificial light–dark cycle with air-conditioning about 22–25 °C and provided with filtered water and standard food. The acclimation time for experimental mice was about 5–7 days before demonstrations. The 5 × 10^6^ KB cells suspended in MEM without FBS were subcutaneous injection in the right hindquarter. The formula 1/2(4π/3)(L/2)(W/2)H was used for calculating the tumor volume (abbreviation: L is the length, W is the width, and H is the height of the tumor). The demonstration was started as the tumors volume about 100 mm^3^. The lyophilized MZF@TPGS was diluted into PBS buffer and filtered with 0.22 μm nylon filter, the iron concentration of filtered MZF@TPGS formulations in PBS solution was analyzed by AAS. The 0.1 mL of MZF@TPGS formulations in PBS solution (concentration: 5 mg Fe/kg) was demonstrated via a lateral tail vein. After treatment, a 1 cm diameter NdFeB magnet with a thickness of 0.4 cm was placed at the tumor site for magnetic fluid guidance, and the magnet was removed after 24 h. MR imaging of the mice was conducted before treatment and 24 h post-injection using a 7 Tesla MRI (BRUKER S-300 BIOSPEC/MEDSPEC MRI, Billerica, MA, USA) while the animals were under halothane gas anesthesia. 

For *T_1_*-weighted imaging, the pulse sequences were chosen TR/TE = 550 ms/10.5 ms and matrix size = 256 × 128. For *T_2_*-weighted imaging, the TurboRARE-T_2_ pulse sequences was selected TR/TE = 5000 ms/56 ms, Flip angle = 180°, matrix size 256 × 128. The FOV was 9 × 3.5 cm for coronal scanning with the slice thickness 1 mm and a 1 mm gap per image. Each scan at a number of excitation (NEX) of 5 with the total scanning time 13 min and 20 s. The MR images were chosen from the tumor area, and approximately 5 sections were analyzed using Image J software, from the NIH (http://rsbweb.nih.gov/ij/) (accessed on 1 January 2015).

### 2.6. Antitumor Efficacy of MZF@TPGS Formulation-Mediated Hyperthermia

In this study, all processes of in vivo experiments were approved by the Institutional Animal Care and Use Committee of National Chung Hsing University (IACUC of NCHU), the ethic approval number: IACUC No. 102-127^R^. After 5–7 days acclimation time for experimental mice, the mice were injected subcutaneously in the right and left hindquarters with 5 × 10^6^ KB cells suspended in 50 μL of MEM without FBS. The tumor sizes and body weights were measured every 3 or 4 days for the duration of the experiment (17 days). The formula 1/2(4π/3)(L/2)(W/2)H was used for calculating the tumor volume (abbreviation: L is the length, W is the width, and H is the height of the tumor). Demonstrations started as the tumors volume nearly 100 mm^3^, which was designated as day 0.

Mice were randomized into different groups for the treatments (PBS-control and intravenous injection group), with three to four mice in each group. The mice were given PBS and MZF@TPGS (5 mg Fe/kg) on day 0, and placed NdFeB magnet as described above. After 24 h, the mice were placed in the AC coil, and the function generator was used to create an applied frequency of 100 kHz and approximately 20 Oe for 30 min on day 1. The tumor size and the change in body weight of each mouse were recorded.

### 2.7. Histological Analysis

The tissues of tumor and liver were excised and embedded in paraffin as the mice sacrificed after MR imaging and hyperthermia treatment. The paraffin embedded tissues were sectioned with thickness of 2 μm for following histological analysis. The sections were de-paraffinized, dehydrated and reacted with hematoxylin and eosin (H&E) for H&E staining [29]. To observe the MZF formulations using Prussian blue after studying in vivo MR imaging and hyperthermia-mediated tumor suppression, the sections were reacted with the equal volume of 20% (*w*/*w*) hydrochloric acid and 10% (*w*/*w*) potassium ferrocyanide for 30 min. The light microscope equipped with a digital camera was used for observing the Prussian Blue stained sections.

## 3. Results

### 3.1. Characterization of MZF Nanoparticles and Their Formulations

There are various approaches can be used for the synthesis of MZF nanoparticles and the synthetic procedure is important for the physical and chemical properties of MZF nanoparticles, such as the composition, particle size and magnetic performance. In this study, we successfully synthesized MZF nanoparticles using high-temperature thermal decomposition method. Comparing with MZF with particle size around 5–20 nm reported previously using similar approach or other synthetic processes (e.g., ceramic technique, hydrothermal, sol-gel or co-precipitation). In terms of size dispersion and uniform morphology, applied synthetic protocol in this study showed superiority over co-precipitation [30], chemical sol-gel combustion [31], or hydrothermal [19] synthesis routes. The MZF synthesized by thermal decomposition by acetyacetonate metal precursors with less surfactants may more uniform shape than the acetylacetonate with chloride salts [32].

**Figure 1:**
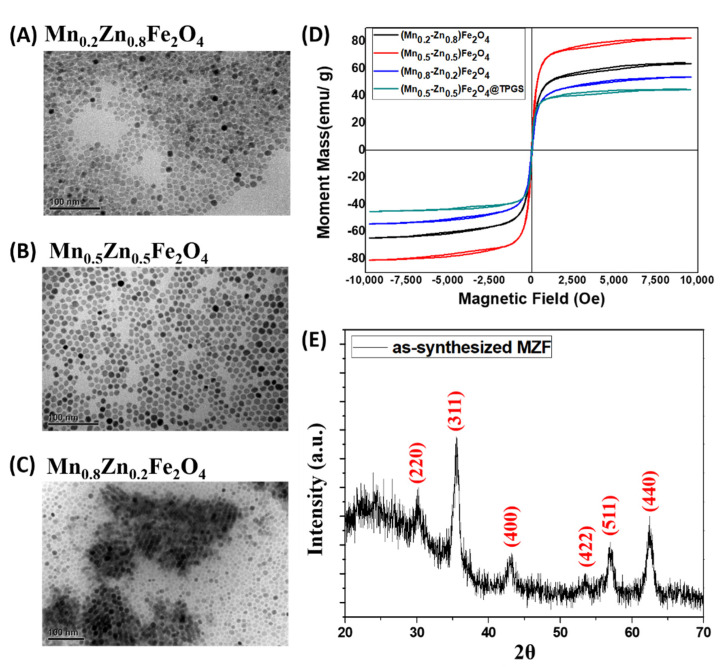
Characterization of the as-synthesized MZF. The TEM images of (**A**) Mn_0_._2_Zn_0_._8_Fe_2_O_4_, (**B**) Mn_0_._5_Zn_0_._5_Fe_2_O_4_, and (**C**) Mn_0_._8_Zn_0_._2_Fe_2_O_4_. (**D**) The hysteresis loops of different Mn/Zn ratio MZF nanoparticles (black: Mn_0_._2_Zn_0_._8_Fe_2_O_4_, red: Mn_0_._5_Zn_0_._5_Fe_2_O_4_, blue: Mn_0_._8_Zn_0_._2_Fe_2_O_4_, green: Mn_0_._5_Zn_0_._5_Fe_2_O_4_@TPGS) and TPGS formulated Mn_0_._5_Zn_0_._5_Fe_2_O_4_ nanoparticles formulations (MZF@TPGS). (**E**) The XRD pattern of as synthesized Mn_0_._5_Zn_0_._5_Fe_2_O_4_ nanoparticles.

The treated Mn^2+^ and Zn^2+^ cation concentration of MZF nanoparticles (Mn_x_Zn_1−x_Fe_2_O_4_) in synthesis process was systematically controlled in x = 0.2, 0.5, 0.8, respectively. The treated Mn^2+^ and Zn^2+^ cation concentration of MZF nanoparticles (Mn_x_Zn_1−x_Fe_2_O_4_) in synthesis process was systematically controlled in x = 0.2, 0.5, 0.8, respectively. The theoretical stoichiometry ratio of MZF (Mn_0_._5_Zn_0_._5_Fe_2_O_4_) nanoparticles were analyzed by AAS and the synthesized MZF were calculated with the composition in Fe 49.95%, Mn 7.19%, and Zn 6.35%. The measured Mn_0_._37_Zn_0.27_Fe_2.36_O_4_, different from theoretical/expected Mn_0_._5_Zn_0_._5_Fe_2_O_4_, might be caused by existed surfactants of oleic acid and oleylamine after the process of vacuum dried. Similar results were previously reported for the introduction of Mn and Zn doping in ferrite system [5,33,34]. TEM analysis was carried out to observe the morphology of the MZF nanoparticles via real-space imaging. The MZF nanoparticles (Mn_x_Zn_1−x_Fe_2_O_4_, x = 0.2, 0.5, and 0.8) were verified by TEM and had sizes of 12.97 ± 1.97 nm, 10.35 ± 1.25 nm, and 11.34 ± 1.70 nm, as analyzed by SigmaScan Pro statistics software and shown in Figure 1A–C without obviously differences in sizes performance between different Mn/Zn ratios. Through this method, the synthesized MZF were easily monodispersed in smaller sizes with well magnetic performances compared to other synthetic strategies. The morphological characterization of the particles indicated that they were almost uniform in size around the average particle size [22,35].

To evaluate the magnetic performances of these MZF nanoparticles, the as-prepared MZF and MZF@TPGS formulations were evaluated by VSM sweeping between approximately −10 and 10 kOe at room temperature (nearly 300 K). The saturation magnetization (M_s_) values of the as-synthesized MZF nanoparticles with different Mn/Zn ratios and MZF @TPGS formulations were shown in Figure 1D. The Mn_0_._5_Zn_0_._5_Fe_2_O_4_ nanoparticles had the highest M_s_ value of a 82.63 emu/g, and the M_s_ values of Mn_0_._2_Zn_0_._8_Fe_2_O_4_ and Mn_0_._8_Zn_0_._2_Fe_2_O_4_ are 64.33 emu/g and 45.20 emu/g. The oleic acid/oleylamine-stabilized Mn_0_._5_Zn_0_._5_Fe_2_O_4_ nanoparticles had a small coercivity (3.31 G). The magnetic performances may have differed from other studies due to the synthesis process, compositions, particle sizes, and other detail conditions. Our prepared MZF nanoparticles revealed particle size around 10–13 nm with higher magnetization and lower coercivity, [25,33] facilitating for MR contrast applications. The oleic acid/oleylamine-stabilized Mn_0_._5_Zn_0_._5_Fe_2_O_4_ nanoparticles is used for followed experiments due to its higher magnetization with lower coercivity. The magnetic moment and M_s_ affected by Mn^2+^ cation concentration which resulted from the replacement of Zn^2+^ ions and/or the development of antiferromagnetic alignment of Fe^3+^ ions [25]. The prepared MZF were successfully doped with Mn and Zn; however, its saturation magnetization was smaller than the reported values of the bulk iron oxide (M_s_ value about 92–100 emu/g) [36] or other reported MZF particles [37,38]. The possible reasons may be due to the smaller size with the surface effects, compositions or magnetically distorted surface layer. The surfactants coated also influenced on the magnetic properties through alteration of surface spin of magnetic nanoparticles positively or negatively [39,40]. The M_s_ and the coercivity of the MZF@TPGS formulations coposited by Mn_0_._5_Zn_0_._5_Fe_2_O_4_ nanoparticles are 45.00 emu/g and 2.32 G. Though the M_s_ decreased after formulated with TPGS but the coercivity decrease slightly. In our previous study, cluster-like magnetic particle formation may have slightly enhanced the superparamagnetic performance for MR contrast agent and increased the specific absorption rate (SAR) value in electromagnetic hyperthermia applications compared with previous reports [26,41,42]. The CuKα X-ray powder diffraction patterns of the synthesized MZF nanoparticles are shown in Figure 1E. The X-ray diffraction peaks corresponded to the spinel structure of MZF [25,43,44,45].

**Figure 2:**
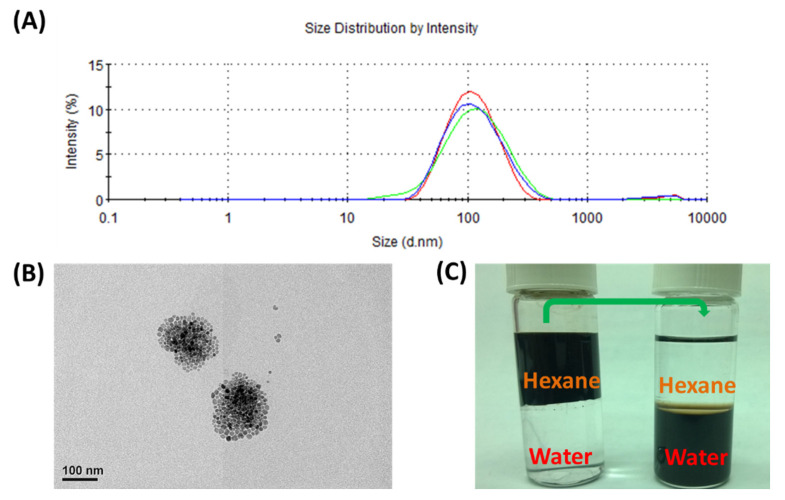
(**A**) The size distribution and (**B**) a TEM image of the MZF@TPGS formulations. (**C**) Photograph of as-synthesized MZF dispersed in hexane and modified MZF@TPGS in an aqueous.

The preparation of the MZF formulations using non-ionic and biocompatible polymers of TPGS was followed by an emulsion and solvent evaporation approach [26]. The mixture solution was heated with a digitally controlled heating mantle at approximately 80–85 °C for 20 min, the MZF@TPGS was prepared after evaporation of the hexane solvent. The size distributions of MZF@TPGS were evaluated by DLS. In addition, the results evaluated by DLS indicate the size distributions of the emulsified bodies and the stable nanoformulation with narrow size distributions after the preparation process. In Figure 2A, the average hydrodynamic size and size distributions average of MZF@TPGS were approximately 98.94 ± 0.835 nm with a polydispersity index (PDI) of 0.226 ± 0.007 evaluated by DLS, and the size observed by TEM was 153.39 ± 17.52 nm. 

The strong interdigitated hydrophobic interactions from the hydrophobic Vitamin E functional groups of TPGS and the alkyl chains of the oleic acid resulted in stable spheres [46]. In the process of hexane evaporation, the mixed micro oil droplets of hexane, oleic acid stabilized MZF nanoparticles, and the surfactant TPGS had condensations and formed the MZF@TPGS formulations in the phase-transfer process. This formation process may be due to the lower boiling point of hexane as an oil phase than water that could be easily removed by solvent evaporation. The full passivation of the surface of the formulations by TPGS improved the water solubility of MZF@TPGS formulations and the hydrophilic polyethylene glycol (PEG) chains of TPGS were spread on the formulation surface and provided negative charge to stabilize the MZF formulations which prevented the agglomeration between the clusters in the aqueous solution. 

### 3.2. Relaxivity, MR Imaging and Hyperthermia Tests of MZF@TPGS Formulations

**Figure 3:**
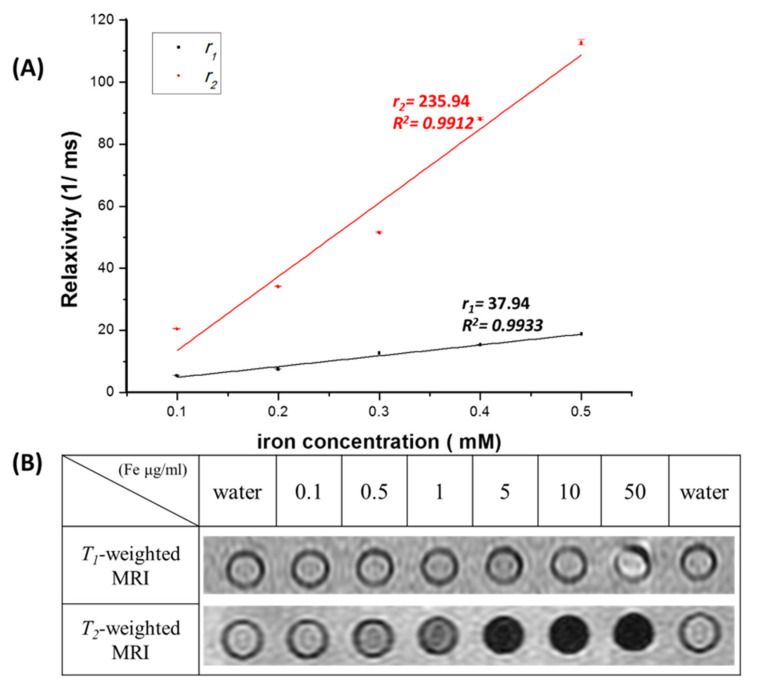
(**A**) *T_1_*-weighted and *T_2_*-weighted relaxivity plots measured by a pulsed NMR Minispec. (**B**) MR images of the MZF@TPGS formulations taken by a 3 T clinical MR scanner.

Commonly, the superparamagnetic particles can performed as negative MR contrast agents due to the ability of shorten the proton relaxation time of spin-spin (*T_2_*) and resulted in *T_2_*-weighted images by signal reduction and darkness [47,48]. To evaluate the MR contrast ability of MZF@TPGS formulations as an innovative dual potential MR contrast agent, the *T_1_-*weighted and *T_2_-*weighted relaxation times were measured by a 20 MHz (0.47 T) Minispec at 37 °C. Figure 3A shows the values *1/T_1_* and *1/T_2_* and the relaxivity constants of *r_1_* and *r_2_* were calculated from the slopes of the linear plots of the relaxation rates against the iron concentration. The dual MR imaging contrast ability may be altered under different magnetic fields. It is well known that most of the magnetic materials that behave as dual contrast agents at low magnetic field, actually lose this capability at high field, the relaxivities in different micro-environment would be changed significantly. In this study, the MZF@TPGS formations revealed almost 3-fold *r_1_* value (37.94 s^−1^ mM Fe^−1^) compared with that of iron oxide@TPGS (r*_1_* value 12.37 s^−1^ mM Fe^−1^)) whereas the *r_2_* value of the MZF@TPGS formulation and iron oxide@TPGS were similar (235.94 vs. 253.85 s^−1^ mM Fe^−1^). The MZF@TPGS formulations achieved both higher *r_1_* and *r_2_* values with an *r_2_*/*r_1_* ratio of 6.219 compared with the commercial product Resovist^®^ (*r_2_*/*r_1_* = 5.98, r*_1_* value 25.40 s^−1^ mM Fe^−1^, r*_2_* value 151.95 s^−1^ mM Fe^−1^), based on the results of our previous report [26]. According to our results and previous studies, MZF and iron oxide nanoparticle solutions can be emulsified and stabilized by TPGS successfully than can reduce the surface tension of oil droplets by aligning at the interface between oil and water, the M_s_ value of TPGS formulated magnetic nanoparticles would decrease but the *r_2_* value increase due to the hydrophilic material stabilized [26]. Qiu et. al. reported the properties of individual and clustered iron oxide nanoparticles, including magnetization, magnetic moments, and contrast enhancement in MRI. The magnetic enhancement from cluster-liked formulations may due to their collective properties, the clusters were more responsive to the external magnetic field and can potentially serve as better MR imaging contrast enhancement agents than individually dispersed magnetic nanoparticles in relatively lower iron concentration. There were only lightly changes of coercivity of clusters compared to individual ones was observed. This was likely due to the presence of both anisotropic and random dipole−dipole interactions within the clusters, which makes cluster behave like a non-interacting system. The phase exchanged liquid was homogenized under magnetic stirring through the entire process, the total formulations were magnetized, which could have introduced some anisotropy to the assembly through the partial alignment of magnetic moments of these magnetic nanoparticles [42]. This finding indicates that the TPGS-stabilized MZF formulation can not only perform well as a *T_2_*-weighted contrast agent but can also potentially act as a dual *T_1_*-weighted and *T_2_*-weighted contrast agent. To further confirm the MR contrast agent ability of the MZF@TPGS formulations, a 3 T clinical MR scanner was used for evaluating the *T_1_*-weighted and *T_2_*-weighted MR contrast ability of MZF@TPGS with different iron concentrations. In Figure 3B, it can be seen that the MZF@TPGS formulations exhibited a signal intensity reduction with concentration increase in the *T_2_*-weighted MR images. In the *T_1_*-weighted MR images, the MZF@TPGS formulations did not show obviously contrast enhanced at lower concentrations, the brightness contrast enhancement of MZF@TPGS formulations can be observed at 50 μg Fe/mL. The *T_1_*-weighted MR images of Resovist^®^ showed brightness contrast ability about 5–10 μg Fe/mL but turned into darkness contrast enhancement significantly in the 50 μg Fe/mL (data not shown). The applications of iron oxide-based nanoparticles as MR contrast agents are limited by the higher *r_2_* value at large *r_2_/r_1_* ratio. To realize innovative uses of iron oxide-based nanoparticles as dual MR contrast agents, the strategy is to increase the *r_1_* value of contrast agents with small *r_2_* value or, alternatively, to increase the *r_1_* value as large as possible [49]. The transfer of the MZF into water soluble MZF@TPGS, the formation of cluster-like could possibly be related to several physicochemical factors such as water molecules interactions, poor hydrophilic/hydrophobic balance, and surface charge density among others, also it is important to take into account The protonation and the polymer hydration state and consequent swelling. The clustering effects of both TPGS formulations indeed cause high *r_2_* value compared with Resovist^®^. The increased *r_1_* value of MZF@TPGS formulation may be due to the protonation and hydration of polymer, thereby resulting in the entry of water molecules into cluster-like area, the interacting with the metal of Mn and Zn, shortening the relaxation time and giving rise to positive or negative contrast on *T_1_*- or *T_2_*-weighted magnetic resonance images [50]. Compared to reported silica-coated MZF core [51] or worm-like formulated MZF [52] with higher *r_2_* value for *T_2_*-weighted MR contrast, our prepared MZF@TPGS formulation revealed dual MR contrast ability due to the balance in *T_1_*- or *T_2_*-weighted imaging.

The hyperthermia coil system of the equipment may have demonstrated near by the skin. The general skin temperature is about 32–35 °C when the air is still. The controlled temperature 32 °C as a started temperature is followed by the USP<724>Drug release and USP<1724> Vertical Diffusion Cell. The temperature equilibrates to 32 ± 0.5 °C during the evaluations of specific skin test. In the evaluations of hyperthermia test, the started temperature 32 °C was only controlled at start but did not maintained due to the limitations on the devices. The hyperthermia test of the MZF@TPGS formulations at a concentration of 0.4 mg Fe/mL was performed in an AC solenoid coil-capacitor system under 100 kHz and approximately 10 Oe controlled by a function generator for 30 min, and the temperature raised from 32 °C to 37.8 °C. The applied field (*H*) of 10 Oe and a frequency (*f*) of 100 kHz are in the biologically safe and physiologically tolerable range with lower *Hf* (7.958 × 10^7^) compared to previously studies. The high field strengths may produce damage to tissue through eddy current heating. The ideally magnetic materials for hyperthermia should have a very high SAR at lower field strength with specific frequency is practically suitable for the hyperthermia treatment. The SAR value was calculated using the following equation: SAR = ΔT/Δt ⋅ msample/mMZF@TPGS ⋅ cwater, with total sample mass as msample, mass of Fe in the MZF@TPGS as mFe, and the heat capacity of water c as 4.19 J/g K. (ΔT = 37.8 − 32 = 5.8 °C, Δt = 1800 sec, msample = 2 g, mFe = 0.8 mg (0.4 mg Fe/mL), cwater = 4.19 J/g K, SAR = 33.753 W/g) This value was considered to be lower than the magnetic material specific used for hyperthermia. In the report from K. Kekalo et. al., they compared the SAR value (W/g Fe) of different materials under different magnetic field strength (Oe), the report showed the SAR value may lower than 50 W/g Fe under the filed strength lower than 100 Oe [22,25,53,54]. Heat dissipation from magnetic materials is caused by a delay in the relaxation of the magnetic moment through Néel’s relaxation (rotation within particles) and Brownian relaxation (rotation of the particles themselves) [55]. The physical nature of AC magnetically induced heating of solid-state soft ferrite magnetic materials is thought to be predominantly related to the relaxation loss heating power, *P*_relaxation loss_, as described in previous reports [53,56]. The magnetic properties of the MZF@TPGS formulations shown in Figure 1D indicate low coercivity and superparamagnetism, but the performance in the hyperthermia test showed that the MZF@TPGS formulations can also potentially be used for electromagnetically induced hyperthermia for cancer treatment at lower amplitude.

### 3.3. In Vitro Hyperthermia Efficacy of MZF@TPGs Formulation in KB Cells

**Figure 4:**
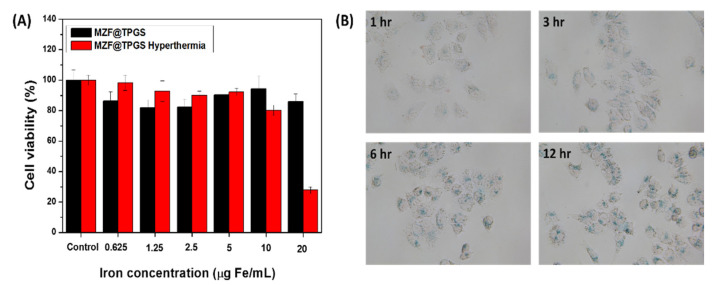
(**A**) Viability of KB cells evaluated by the MTT assay after the treatments with the MZF@TPGS formulations and after hyperthermia. The results are presented as the viability means ± standard deviations. (*n* = 3) (**B**) The KB cells cellular uptake of the MZF@TPGS formulations in at 37 °C from 1 to 12 h, which was evaluated by Prussian blue staining and visualized by optical microscopy with digital camera.

The cytotoxicity of the MZF@TPGS is an important issue that needs to be addressed before they can be applied in the studies in vitro and in vivo. The cytotoxicity of the MZF@TPGS formulations without and with hyperthermia toward KB cells was determined using the colorimetric MTT assay. For this test, KB cells were incubated with the MZF@TPGS formulations in different concentrations for 24 h, after which the viability was quantitatively analyzed. The started temperature 32 °C was controlled at beginning before the in vitro hyperthermia test. As shown in Figure 4A, the viability of KB cells revealed almost no obvious toxicity to the cells at an iron concentration of 20 μg Fe/mL. The cytotoxicity is based on the both outer stabilizers and the inner core materials. The MZF@TPGS formulations were stabilized by the non-ionic surfactant TPGS, which can prevent cytotoxicity of ionic surfactant and larger aggregates [26]. 

Like other metal based materials, the cytotoxic and genotoxic effects of metallic nanoparticles mainly attributed to their ability to produce reactive oxygen species (ROS) [57]. Heavy metals are the one of key regulators in cell apoptosis. The metals with affinity to the thiol-group containing enzymes and proteins which relative to cellular defense mechanisms [58]. Mn is a constitutive element of a series of enzymes and cofactors fundamental for neurologic function, such as superoxide dismutase, glutamine synthetase and others. Mn acquired from the diet, drinking water or as inhaled particles may be assimilated and delivered to the neuro system [59]. In the previously reports, there was no significant decrease in the viability of the HeLa cells observed in the report of Hong Yang et al., for superparamagnetic manganese ferrites at concentrations below 200 μg/mL [60]. Jian Lu et al., reported that manganese ferrite nanocrystals synthesized in an organic phase and stabilized by mPEG-b-PCL had no obvious cytotoxicity on the HepG2 and RAW 264.7 cell lines [61]. In the MR imaging research, G. W. Yang et al., demonstrated the Mn-based nanoparticles are safe and effective MR contrast agent for in vivo imaging base on the in vitro and in vivo assessments of biocompatibility, especially the evidence of immune toxicity [62]. These reports indicated the potential applications of Mn based nanoparticles for MRI as safe contrast agents for in vivo imaging. Zinc based nanoparticles are one of the promising metal oxide nanoparticles which are used in industrial products including cosmetics sunscreens, medical materials and others. The potential risk of Zn based materials exposure to humans increased sharply in recently years. Parimal Karmakar et al., reported the spherical ZnO nanoparticles showed no obviously toxic effect in their observations. There are several reports demonstrating Zn based nanoparticles as non-toxic materials in different human cell lines [63]. Zinc ferrite nanoparticles have low toxicity from zinc ions compared with other metallic cations. Jiaqi Wan et al., evaluated the cytotoxicity of zinc ferrite nanoparticles and suggested that they possess a good safety profile [64,65]. These researches demonstrated the evaluations of safety in the Mn and Zn based nanoparticles for potential applications.

Magnetic hyperthermia can perform as a minimally invasive and promising cancer thermotherapy that produces local heat via the magnetic materials under AC electromagnetic fields. In the hyperthermia test of the MZF@TPGS formulation, the highest concentration of 20 μg Fe/mL resulted in obvious inhibition of cell viability following 24 h incubation after the hyperthermia treatment. The group of control (red bar, cells without MZF@TPGS with hyperthermia) compared to the group of control (black bar, cells without MZF@TPGS without hyperthermia) is about 96.88% (data not shown), the results indicated the cell viability without significantly changes after hyperthermia treatment. However, there were no obvious effects at low concentrations, possibly due to the low amount of heat generated in the cells at lower amplitude applied field (10 Oe). Hyperthermia has been shown to have promising antitumor effects for various types of malignant tumors. The range of hyperthermia temperature (40–43 °C) induces cell killing depending on the temperature and the period of treatment. Till now only two distinct forms of cell death, apoptosis and necrosis, induced by hyperthermia can be recognized morphologically. Hyperthermia treatment activated the ER stress and necrosis as the temperature over 43 °C, serum cytokine analysis revealed that hyperthermia at lower temperature induces an intratumoral inflammatory cytokines and chemokines to increase in enhanced T-cell trafficking. This inflammatory cytokines and chemokines act at multiple discrete steps that favor lymphocyte infiltrate to the tumor microenvironment and attack solid tumors in the immune cascade. However, most of the research was conducted under physiological temperature stress of lower than 40–41 °C to prevent directly necrosis, but the cytotoxic effect of heat stress, mitochondrial heat-induced alterations, metallic ROS, as well as heat shock protein (HSP) expression may activated in the in vitro cellular mitochondrial microenvironments [66]. 

Cellular uptake of the MZF@TPGS formulations was carried out by the Prussian blue to visualize the localization of iron under the microscopy with digital camera. The KB cells were incubated with the formulations for 1 to 12 h, followed by Prussian blue staining. At a concentration of 20 μg Fe/mL of MZF@TPGS formulations treatment, a time-dependent intracellular accumulation was observed in the KB cells shown in Figure 4B. Thus, the TPGS can stabilize the hydrophobic MZF nanoparticles as a hydrophilic MZF@TPGS formulations that can serve as theranostic agents without aggregation and significant toxicity.

### 3.4. MZF@TPGS Formulation-Mediated MR Imaging and Hyperthermia In Vivo

**Figure 5:**
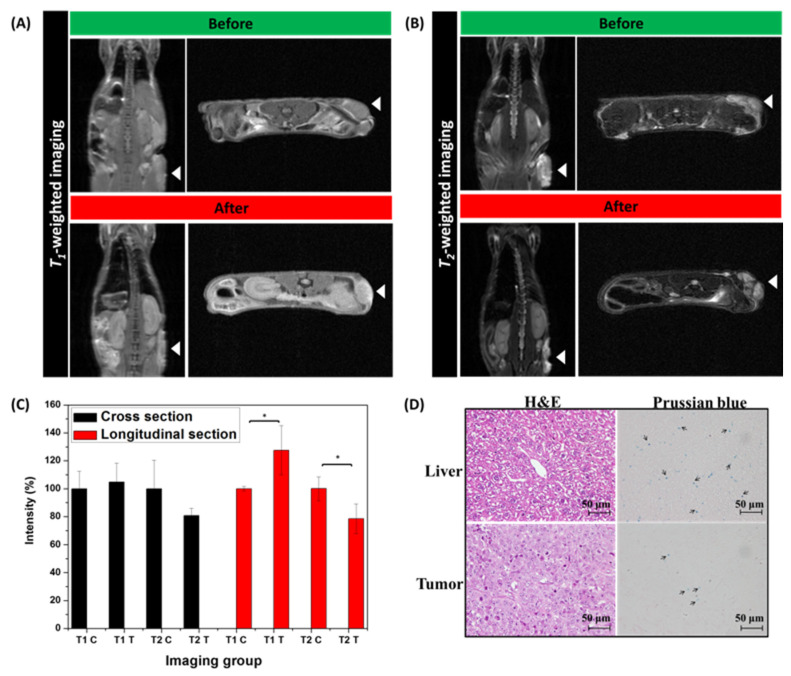
In vivo studies of KB tumor-bearing mice before and 24 h after administering of MZF@TPGS formulations. (**A**) *T_1_*-weighted and (**B**) *T_2_*-weighted animal MR images taken by a 7 T animal MR scanner. (**C**) Analysis of the photo brightness intensity using ImageJ. (* *p* < 0.05) (**D**) The images of H&E staining and Prussian blue staining of liver and tumor sections from the KB tumor-bearing mice 24 h. The arrows indicate iron staining.

To further evaluate the MR contrast ability of the MZF@TPGS formulations in vivo, KB tumor-bearing mice were scanned before administration vs. 24 h post intravenous injection. After injecting the MZF@TPGS formulations, a small NdFeB magnet that was 1 cm in diameter and 0.4 cm thick was placed at the tumor site for magnetic fluid guidance [67]. As shown in Figure 5, the signal intensity of the tumor at the right flank (Figure 5A, white arrow) both changed markedly under *T_1_*-weighted and *T_2_*-weighted imaging at post injection vs. before intravenous injection. The commercial product Resovist^®^ is used for liver tumor imaging due to the accumulation in the reticuloendothelial system (RES) of the normal liver and enhances contrast in liver tumors. In this study, signal intensity was decrease in the liver after the administration of MZF@TPGS formulations (Figure 5B). This finding expresses the activity of macrophages phagocytosis in the liver, which has been exploited and similar with the mechanism for the detection of liver tumors with Resovist^®^. The MZF@TPGS formulations performed an obviously contrast enhancement in both tumor region and in the liver under *T_2_*-weighted imaging. In addition, the *T_1_-*weighted MR contrast enhancement was more obvious in the in vivo study than in the in vitro study. Through image analysis, the photo brightness intensity was significantly different, as shown in Figure 5C. In the *T_1_*-weighted MR images, the tumor site appeared brighter after injecting the MZF@TPGS formulations in both cross section and longitudinal section, but especially in the longitudinal sections of the tumor area. The *T_2_*-weighted MR images provided strong contrast enhancement not only in the tumor area but also in the liver site. These data show that the MZF@TPGS formulations provide strong contrast enhancement both in *T_1_*-weighted and *T_2_*-weighted MR imaging and also function as responsive materials in magnetic fields for magnetic fluid applications. 

After MR imaging, sections of liver and tumor tissue were stained by H&E, and representative images are shown in Figure 5C. Glycogen infiltration was observed in the normal liver, which is due to the in vivo experimental procedure without starvation. Clearly, no noticeable morphological differences were observed both in the liver or tumor after the administration of MZF@TPGS formulation. To verify the accumulation of the MZF@TPGS formulation in the region of liver and tumor sections, the tissue sections were stained by the Prussian blue staining to detect iron-based ferrite MZF. The positive staining in blue color was observed in both the liver and tumor region shown in Figure 5D. It is noticed that abundant PB-stained area exhibited in liver than the tumor, which indicates that the accumulation of the MZF@TPGS formulation in the liver was higher than in the tumor in the 24 h. 

This result was also found in the *T_2_*-weighted MR imaging, as shown in Figure 5B, and was possibly due to the small external magnetic field that only provided limited magnetic fluid guidance in vivo. Dual MR imaging can provide both *r_1_* and *r_2_* relaxivity for calculating signal statistics and for determining both *T_1_* and *T_2_* from one MR image.

**Figure 6:**
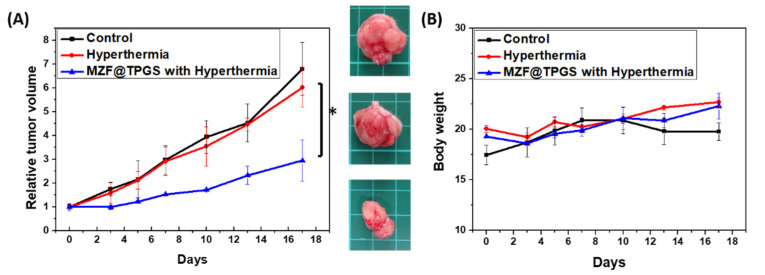
In vivo antitumor effects studies of MZF@TPGS formulations in KB tumor-bearing mice. (*n* = 3 in control, *n* = 4 in hyperthermia, *n* = 5 in MZF@TPGS with hyperthermia) (**A**) Tumor size and (**B**) body weight of the different groups over the experimental period of 14 days (* *p* < 0.05).

The efficacy of MZF@TPGS for inhibiting tumor growth by hyperthermia treatment in vivo was further evaluated. The iron dose (5 mg Fe/Kg) was the same with the MR imaging in vivo administrated in this evaluation. The KB tumor cell-bearing mice were placed into three groups consisting of control (*n* = 3) and IV (intravenous) treatment groups (*n* = 3), and the treatments were performed by IV injections of PBS and MZF@TPGS dispersed in PBS followed by magnetic fluid guidance for 24 h by placing a small NdFeB magnet that was 1 cm in diameter and 0.4 cm thick at the tumor site. The mice, which were anesthetized by an intraperitoneal injection, were placed in a magnetic induction coil one-by-one. The AC solenoid coil-capacitor system (100 kHz with amplitude approximately 20 Oe) was controlled by a function generator to apply hyperthermia treatment for the entire 30 min. After the single hyperthermia treatment, the volume change in the tumor and the weight change of the mice were monitored over a period of 17 days, and the results are shown in Figure 6A,B. The size of the tumor decreased at first with slowly growth rate during 17 days, but the tumor tissue growth slowly possibly due to only a single treatment was applied under relative lower amplitude applied field. It is remarkable that the MZF@TPGS formulations exhibit tumor growth suppressions even when the frequency and the magnetic field amplitudes are considered safe and relative lower compared with other studies at approximately 20 Oe (1591.55 A/m) and *f* = 100 kHz, when the max H*f =* 1.59 × 10^8^ A m^−1^s^−1^. Moreover, hyperthermia alone seems insufficient for tumor cell regression for the result that single treatment of lower temperature hyperthermia itself could not effectively promote the anti-tumor response [41,68]. Many different values of the applied magnetic field can be chosen if works at lower frequencies such that the H*f* factor is below the threshold. Working at lower magnetic field amplitude provides more safe therapeutic environments and the preventions of non-therapeutic tissues area heat generations. This study demonstrated a multifunctional approach for producing dual MR contrast agents and electromagnetically induced hyperthermia at lower applied field (20 Oe), the success of which has been validated using both in vitro and in vivo evaluations. 

On the other hand, the potential applications in physiological ferroptosis had been studied for recent years. Ferroptosis is an iron-dependent form of programmed cell death triggered by unrestricted lipid peroxidation and subsequent plasma membrane rupture. The vitamin E performed as the ferroptosis inhibitors have the potential to alleviate diseases resulting from ferroptosis such as carcinoma, ischemic perfusion injury, neurodegeneration, and acute renal injury. The TPGS is a vitamin E derivative that had been intensively used for drug delivery systems to enhance drug solubility and increase the oral bioavailability of anti-cancer drugs with selectivity for cancer cell growth inhibition. It may not only be useful as a surfactant or carrier for drug delivery, but may also exert intrinsic therapeutic effects suggesting that it may promote a synergistic interaction with formulated specific chemotherapeutic drugs [69,70]. The TPGS inhibited ferroptosis and performed synergistic interaction potentially in cancer treatments compared with other non-ionic or ionic surfactants. The detail mechanism and interaction needs to be more studied, we will continue to research and study in the magnetic materials with TPGS and ferroptosis. Beola et al., showed that the SAR value decreased after cell internalization, primarily due to the different field conditions. There were correlations between the number of particles and size of the vesicles where the particles are trapped, particularly due to the different energy density per surface area through the vesicles containing the membrane of magnetic materials [71]. In this study, MZF@TPGS may be thought to be trapped in specific compartments such as endosomes [26]. After the hyperthermia treatment, the lysosomal leakage has been shown to initiate apoptosis induced by several stimuli. Lysosomal membrane permeabilization would lead to the release of cathepsins into the cytosol, which would cleave and activate some specific proteins as indicators for other stimuli, although the lysosomal involvement in cell death needs more studies [72]. In contrast, the cell temperature may not increase significantly but the hyperthermia-induced cell death, the apoptotic mechanism was mainly intrinsic.

**Figure 7:**
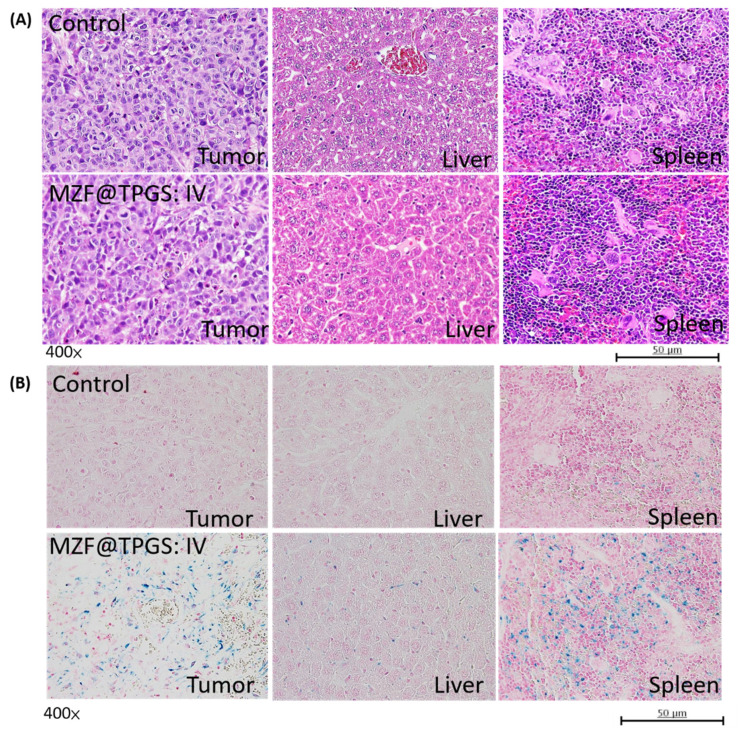
(**A**) H&E staining and the (**B**) Prussian blue staining of tumor, liver and spleen sections from mice after treatment with the MZF@TPGS formulations and hyperthermia.

After 17 days, histopathological assessments of the liver, tumor and spleen were performed, and the results are shown in Figure 7A. No significant changes were observed for the liver and spleen after treatment with MZF@TPGS and electromagnetic hyperthermia. Glycogen infiltration was observed in the normal liver, which was due to the in vivo experimental procedure without starvation. Based on our results, the MZF@TPGS formulations did not influence the morphology of the liver and liver tissue but may cause cell necrosis in the tumor region after the hyperthermia treatment. In Figure 7B, both the liver and the tumor region can be observed positive blue color staining. There were more PB-stained area revealed in the tumor than the liver, indicating that the MZF@TPGS formulation accumulated after 17 days with hyperthermia is different from what was observed with MR imaging 24 h after injection, possibly due to the clearance function of the liver. Compared to liver, there were some MZF@TPGS formulations still accumulated in the spleen may due to the reticuloendothelial system (RES) uptake which the nanoparticle based formulations are generally accumulating in the liver and spleen [41]. 

## 4. Conclusions

In this study, the hydrophobic oleic acid/oleylamine stabilized manganese zinc ferrite nanoparticles approximately 11 nm are synthesized by thermal decomposition using a one-pot synthesis approach with different manganese/zinc ratio. The initial MZF (Mn_0_._5_Zn_0_._5_Fe_2_O_4_) had high saturation magnetization and can be chosen for following studies. The size of TPGS formulated manganese zinc ferrite formulations (MZF@TPGS) is about 100 nm with narrow size distributions. The MZF@TPGS formulations provided higher relaxivity than Resovist^®^ compared with our previous study. It performed as a potential dual MR contrast enhancement with *T_1_*- and *T_2_*-weighted contrast ability both in the evaluations of in vitro and in vivo. Through the magnetic fluid guided, the MZF@TPGS formulations accumulated locally in cancer tissues within the tumor. As the result, in the cancer tissues, both *T_1_* and *T_2_* were significantly decreased and contrast enhanced. Furthermore, application of a safe and lower AC electromagnetic field with approximately 10 to 20 Oe (795.77 to 1591.55 A/m) and *f* = 100 kHz (max H*f =* 1.59 × 10^8^ A m^−1^s^−1^) to tumor bearing mice the day after i.v. injection of MZF@TPGS formulations with magnetic fluid guided significantly inhibited tumor growth. The MZF@TPGS formulations can be demonstrated as a multifunctional cancer theranostic agent for and electromagnetic hyperthermia at lower amplitude magnetic field in both in vitro and in vivo studies. All of the experimental results shown in this study suggest that MZF@TPGS can function as a dual MR contrast agent and may also be useful for hyperthermia treatments.

## Data Availability

Not applicable.
